# A Euclidean Group Assessment on Semi-Supervised Clustering for Healthcare Clinical Implications Based on Real-Life Data

**DOI:** 10.3390/ijerph16091581

**Published:** 2019-05-06

**Authors:** Muhammad Noman Sohail, Jiadong Ren, Musa Uba Muhammad

**Affiliations:** Department of Information sciences and Technology, Yanshan University, Qinhuangdao 066000, China; jdren@ysu.edu.cn (J.R.); musaubamuhammad@stumail.ysu.edu.cn (M.U.M.)

**Keywords:** clustering, consideration analysis, Euclidean assessment, healthcare data, K-mean, projection plot, semi-supervised learning, SOM, visualization, Weka

## Abstract

The grouping of clusters is an important task to perform for the initial stage of clinical implication and diagnosis of a disease. The researchers performed evaluation work on instance distributions and cluster groups for epidemic classification, based on manual data extracted from various repositories, in order to evaluate Euclidean points. This study was carried out on Weka (3.9.2) using 281 real-life health records of diabetes mellitus patients including males and females of ages>20 and <87, who were simultaneously suffering from other chronic disease symptoms, in Nigeria from 2017 to 2018. Updated plugins of K-mean and self-organizing map(SOM) machine learning algorithms were used to cluster the data class of mellitus type for initial clinical implications. The results of the K-mean assessment were built in 0.21 seconds with nine iterations for “type” and eight for “class” attributes. Out of 281 instances, 87 (30.97%) were classified as negative and 194 (69.03%) as positive in the testing on the Euclidean space plot. By assessment for Euclidean points, SOM discovered the search space in a more effective way, but K-mean positioning potencies are impulsive in convergence. This study is important for epidemiological disease diagnosis in countries with a high epidemic risk and low socioeconomic status.

## 1. Introduction

The key findings of data mining algorithms require the computational platform to configure the innovation of databases by performing the predictions and assessments of the implementations [1]. Machine learning establishes the foundation to determine such systems that have the ability to improve the assessment level of results in the required field by practice and knowledge. By recent surveys, the grouping of two technologies (machine learning and data mining) has resulted in the platform of computer science and engineering and solved a wide range of glitches. Basically, data mining technology recycled the systemized data analysis techniques to discover the patterns among the datasets. The three techniques, namely regressions, classification, and clustering, are being used in data mining [2,3,4]. These research findings are dealing with the clustering techniques involved in the data mining phase because it is always extremely important to figure out the similarities in the datasets while dealing with a large number of datasets. It is the assignment of consigning the set of objects into groups, in order to differentiate the objects of one cluster from others. This specific task is involved in many approaches for data analysis, pattern detection, judicial scrutiny of iconic images and information repossession. Clustering is hence called an ample gismo for data analysis in bioinformatics, marketing, scrutiny of images and so on [5,6,7,8]. In addition, many basic queries arise during the research, such as which algorithm should be the best fit on datasets and which algorithm will be used sparingly under which necessary conditions.

In this paper, our focus is on realistic clustering for the Euclidean assessment, when the cluster is present in the dataset. If the visible cluster is not present in the data, then the clustering result will likely move towards the illogical and unclear. To achieve the goal, updated plugins of K-mean and self-organizing map (SOM) algorithms were used on the real-life health data of 281 diabetes mellitus patients. The dataset contains semi-supervised clusters to distinguish mellitus (type 1, type 2 and gestational) from the other chronic disease symptoms. Therefore, this study was performed on the data mining platform Weka 3.9.2(www.cs.waikato.ac.nz) to classify and find the cluster in groups, and to test the Euclidean distance of positive and negative clusters. In addition, this assessment study is important for the initial phase of epidemiological classification of disease diagnosis.

Consequently, the remainder of the paper is arranged as follows: the background of the study is described in the second part of Section 1; Section 2 describes the materials and methods; and Section 3 reviews the results and concludes the outcomes with discussion.

### Background

**Figure 1:**
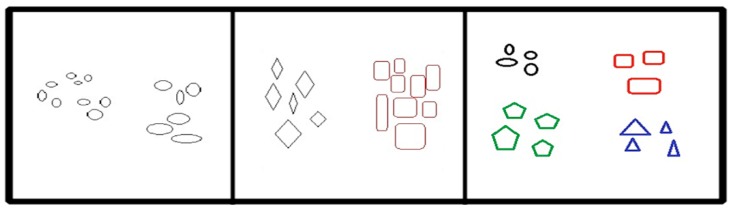
Classic example of a cluster to explain the most moral logic for the cluster distribution.

The aim of clustering analysis should be developed with either the realistic approach or the constructive approach. Figure 1 demonstrates an understanding of cluster constitution, which illustrates the fifteen key points. There are three possible ways to distribute them into distinct clusters. The most dynamic interpretation of these points is to arrange them into two cognitive clusters. Each occupies three agent clusters and is only taken up when the cluster is wisely allowed to nest. Hence, the distribution of two more massive clusters into three substitute clusters may rely on the use of the chromatic scheme. Therefore, it is not arbitrary to state the essential points from specific clusters. Ultimately, we can say that the most satisfactory definition with regards to the analysis of clusters absolutely depends on the data type and the desired results. 

Various researchers have discussed different techniques, for example Marukatat et al. presented the work carried out by Kulis about a technique to generate the Gaussian vectors in Kernal-induced space based on the kernel function [9]. Daniel et al. proposed a work theory on the analysis of Euclidean distance measurements for road distances in Brazil [10]. He mentioned that the distance represents an influential part of the transport cost, which relates to freight. His work aims to develop a procedure to define the diverse factors using statistical tools. Saptarshi et al. worked on a divergence-based system to measure the Euclidean points for the clusters that decide the best proper measures [11]. He recommended a point-to-point distance measure by the S-divergence measures, with the supervised learning scheme in clustering called k-distance impulse method assessment. Raj Bala et al. performed a comparative analysis to measure the Euclidean points in clustering by using four algorithms, namely K-mean, hierarchical, expectation and minimization algorithm, and density-based algorithm [12]. He concluded the results by showing K-mean takes less time to find the accuracy as compared to others. Gaoxia et al. worked on the distance measurement for the time series and stated that measurements are possibly based on the SOM algorithm in the neural networks, which can easily capture the temporal structure of a series [13]. He mentioned some measuring models for the assessment of time-domain and frequency, which named the feature-based measurements. Lai et al. used three algorithms to distinguish the clusters by class namely K-mean, hierarchical and density-based algorithms [14]. By testing, he stated that the density-based algorithm for clustering was insufficient for the high variance density data. K-mean produces reliable results and the hierarchical algorithm was found to be sensitive to noisy data. Gregory Piatetsky stated in the Knowledge Discovery (KDD) conference: “Weka is a landmark system in data mining and machine learning history for the research communities, cause it holds the toolkit that has undoubtedly gained such extensive espousal and survived for a prolonged period. [15]” There was substantial attention paid to the determination of how distinctive clustering techniques were utilized in different areas of the environment [16,17,18,19,20] and in the healthcare sector for different disease predictions [21,22,23,24,25,26,27,28,29,30,31,32]. In addition, K-mean and SOM (self-organizing map) were used in this study for grouping the clusters of the real-life diabetes dataset, after careful analysis by the literature. 

## 2. Materials and Methods

### 2.1. Ethical Consent

The study was approved by the Yanshan University Research Ethics Committee and was performed in accordance with the ethical standards laid down in the 1964 Declaration of Helsinki, as well as its more recent ethical standards. Informed consent was obtained from all individual participants included in the study. 

### 2.2. Method Framework

**Figure 2:**
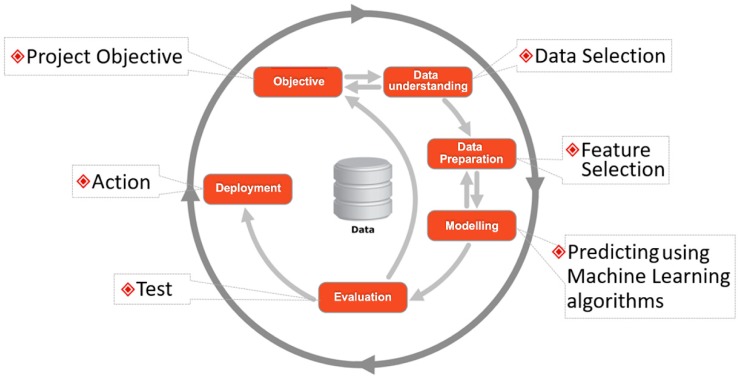
The model strategy used for the assessment of Euclidean groups with machine learning algorithms.

Figure 2 presents the model strategy adopted in this research, which was performed on a data mining platform called Weka by utilizing the machine learning algorithms SOM and K-mean. The method was constructed into six parts, namely data understanding, data preparation, feature selection, modeling, evaluation and results. Moreover, we have aimed to do the following:

(a) Distinguish diabetes mellitus from the other chronic diseases from the dataset, for which we need to establish a two-cluster analysis for positive and negative points.
(b) Efficiently generate four clusters for comprehensive analysis of diabetes mellitus type 1, type 2, gestational, and other chronic diseases.

### 2.3. Data and Questionnaire

Real-life data of 281 diabetes mellitus patients were used in this study, which were collected from the seven largest hospitals in Nigeria, including patient flow, namely Abdullahi Wase Hospital, 36 patients (12.81%); Ajingi General Hospital, 22 patients (7.82%); Federal Medical Center, 56 patients (19.92%); Gaya General Hospital, 28 patients (9.96%); Murtala Specialist Hospital, 88 patients (31.31%); Jidda General Hospital, 20 patients (7.11%), and Sansui General Hospital, 31 patients (11.03%). The questionnaire was designed by consulting the doctors and medical specialists with 108 medical features. The data obtained were from July 2017 to July 2018 including males and females of ages>20 and <87, who were also experiencing symptoms of other chronic diseases.

### 2.4. Attribute Characteristics

The real-life data contain various medical features, such as age of the patient, gender, glucose level, body mass index, hypertension status, glycaemia, family cardiovascular history, work stress level, occupation, status of vision disorder, body status, family history status of diabetes, physical exercise, visits to medical specialists in the last 6 months, number of insulin injection intake, and many others. The observational data covered 108 features (attributes/variables), but only the attributes “type”, including type 1, type 2, and gestational, and other chronic diseases status were used for the final Euclidean assessment of clusters and groups.

### 2.5. Data Mining Platform

Waikato Environment of Knowledge Analysis (Weka) was used to conduct the experiment for clustering groups with updated machine learning clustering algorithms, namely K-mean and SOM. The collected data were in paper form, and were carefully analyzed and converted into (.csv) format to run on Weka. 

Instead of using other data mining platforms like Orange, Tangra, and Knime, we adopted an updated version of Weka (3.9.2). An advantage of using Weka is that it avoids overfitting and unnecessary complexity. In addition, its upgraded plugins provide a more adequate analysis of the dataset. In addition, Weka was awarded the ACM SIGKDD service award [33] for the capital developments in its inclusive packages. It holds five applications to conduct the assessments and analysis of the dataset, namely the explorer, experimenter, knowledge flow, workbench, and simple command line interface (CLI). These applications provided extensive support to the experiment including the necessary preparation of data. 

### 2.6. Clustering via Self-Organizing Map (SOM)

Self-organizingmap (SOM) is also known as neuro computational algorithm and its ultimate goal is to positively identify the set of objects with similar characteristics and accurately map the significant dimensional data into the two dimensions of space [34]. This efficient algorithm differs from the other neural network algorithms in the sense of neighborhood functionality. SOM uses neighborhood functions to protect the topologies for the input space and is famous for its nonlinear methods to reduce the dimensionality and conception of valuable data. SOM is most frequently used in the first stage of clustering with the dataset, where it is able to perform an automatic finding of relevant subgroups and clusters. This kind of appropriate methodology is considered as the two levels of clustering methods. Furthermore, the advantage of this effective method is to be able to handle typically a massive set of data.

The parameters selected for SOM were 2000 for ordering epochs, 1000 for convergence epochs, height and width was set as 2, and learning rate was set as 1.0. The algorithm of the SOM works in six periods with the parameters of $\sigma_{0}$, $\in_{0}$ and $T_{max}$. The first stage is called the initializing stage where it randomizes the weight of nodes. Then it selects each instance from the dataset, and the specific instance is processed numerous times. SOM finds the closest nodes for the best unit, which is called the competition phase, and it updates the weight of each node by (Equation (1)) but not with the same degree, which is known as the cooperation stage. Where the weight of the updated node j knows that $j^{*}$ is the winning node, it ensures the resemblance of weights between the contiguous nodes. Where h is a neighboring function and works according to (Equations (2) and (3)), its amplitudes decrease the spatial width of the kernel according to the step index (t). In the last stage, it reduces the intensity of the updates gradually by (Equation (4)) and repeats again.

(1) $$W_{t+1}\left(j\right)=W_{t}\left(j\right)+\varepsilon_{t}*h_{t}\left(j*j^{*}\right)*\left(W_{t}\left(j\right)-x\right)$$

(2) $$h_{t}\left(j,j^{*}\right)=\exp\left(-\frac{d^{2}\left(j,j^{*}\right)}{2\sigma^{2}\left(t\right)}\right)$$

(3) $$\sigma \left(t\right)=\sigma_{0}\exp\left(-\frac{t}{T_{max}}\right)$$

(4) $$\varepsilon_{t}=\varepsilon_{0}\exp\left(-\frac{t}{T_{max}}\right)$$

The SOM learning algorithm initializes with the three specific phases of standard output after the input has been assigned as:

INPUT:

- Data *X* = *X^i^*, where *i* = 1 *to n*
- Self-organized map algorithmic protocol *W^i^*, where *i* = 1 *to m*
- Maximum number of iterations *T_max_*

OUTPUT:

- Partitions in the set of inter-connected units *P* = *C_i_*, where *i* = 1 *to l*
- Value of density associated to each unit *D_i_*, where *i* = 1 *to m*
- Initial phase:
  - Initialize all neighborhood connection values to zero
  - Initialize all values of unit density to zero
- Competition phase:
  - Present all the patterns of input *X^k^*
  - Choose the best and second best match units BMU U*, U** as in (Equations (5) and (6))
    (5) $$U^{*}\left(X\right)=argmin_{1\leq i\leq m}∥X^{k}-W^{i}{∥}^{2}$$
    (6) $$U^{**}\left(X\right)=argmin_{i\neq U^{*}\left(X\right)}∥X^{k}-W^{i}{∥}^{2}$$
- Adoption phase:
  - Update *W^i^* according to the learning rate of *ε*(*t*) and increase the value of density for every unit *i*, as in (Equations (7) and (8))
    (7) $$W^{i}\left(t\right)=W^{i}\left(t-1\right)-\varepsilon \left(t\right)K_{i,U^{*}\left(X^{k}\right)}\left(W^{i}\left(t-1\right)-X^{k}\right)$$
    (8) $$D^{i}\left(t\right)=D^{i}\left(t-1\right)+r\left(t\right)e^{-\frac{∥X^{k}-W^{i}\left(t\right){∥}^{2}}{2\lambda^{2}\left(t\right)}},\;\mathrm{Where}\;r\left(t\right)=\frac{1}{1+e^{\left(-\frac{t}{T_{max}}\right)}}$$

### 2.7. Clustering via K-Mean

The K-mean algorithm simplifies the classification of a dataset through a certain number of clusters [35,36]. The idea behind K-mean is to define the K centroids for each cluster, but these cluster centroids should be placed in a schematized way because of their diverse locations, producing inconsistent results. Therefore, to attain the proper predictions, the centroids should be placed at a certain distance as far away as possible from each other. After that, the algorithm seizes each specific point belonging to the given dataset and associates it to the most adjacent centroids. If there is no specific point, it remains that the first phase of the algorithm process is complete and the primary grouping is properly defined. 

The parameters selected for the K-mean were 100 as maximum canopies to hold, 2.0 for the minimum density of canopies, 10,000 for canopies’ periodic rate, canopy T_1_ was set as (−1.25), canopy T_2_ was set as (−1.0), maximum iteration was set as 500, and the number of execution slots were given as 1 with 10 seeds. By this stage, it recalculates the new K centroids as the barycenter of the clusters as resulted from the first phase. A new bound must be calculated correctly after the K creative centroids, between the points of the same dataset and the most adjacent new centroid. For that, the loop continuously generates a shift on each step of K centroids the centroids do not move anymore. Finally, the algorithms focus on minimizing an objective function with (Equation (9)) structured as:

(9) $$J=\overset{k}{\underset{j=1}{\sum }}\overset{m}{\underset{i=1}{\sum }}∥x_{i}^{\left(j\right)}-c_{j}{∥}^{2}$$

where m is the number of data points in the i clusters and k is the number of cluster centers, and $∥x_{i}^{\left(j\right)}-c_{j}∥$ represents the Euclidean distance between $x_{i}^{\left(j\right)}$ and $c_{j}$.

First, place the K points into the considerable space as represented by the objects that are being clustered. These essential points indicate the initial group of centroids. Second, assign each object to the group that possesses the most adjacent centroid. After the assigning of all the objects, recalculate the prominent position of the K centroid. Repeat until the centroids are not able to move anymore. This efficiently produces the possible separation of groups objects, for which the matrix to be minimized can be accurately calculated by (Equations (10) and (11)).

(10) $$argmin_{c_{j}\in C}\;dist{\left(c_{i},x\right)}^{2}$$

(11) $$C_{i}=\frac{1}{\left|S_{i}\right|}\sum_{X_{i}\in S_{i}}X_{i}$$

## 3. Results 

Weka (3.9.2) optimizes an “auto Weka” classification for the initial classification of the dataset, by utilizing the best-incorporated filters for the distribution of the training and testing dataset. In our case, “auto Weka” used the “randomize” filter with the 10-fold cross-validation on the testing dataset with the best-fitted classifier “AdaBoost M1” of mean accuracy 98.73% along with average error of 0.001%. In addition, we adopted the updated plugins of K-mean and SOM clustering algorithms for our extensive research on the real-life dataset of diabetes patients of ages>20 and <80 including both males and females, and we correctly classified the clusters by two possible ways. One is by specific diabetes type to separate the mellitus types from other chronic diseases, and the second is by a privileged class of positive tested and negative tested points from the given dataset. This section will demonstrate the satisfactory results of clustering graphically so that readers can understand more clearly, along with the projection plot of positive tested and negative tested clusters.

### 3.1. K-meanAssessment

After examining the Weka by the 10-fold cross-validation, we applied theK-mean algorithm to the dataset to carry out the experiment on the two attributes “type” and “class.” The resulting model builds in 0.21 second with the considerable number of a total of nine iterations on the diabetes dataset “type” attribute and eight iterations on the diabetes dataset attribute “class”. The final results for the K-mean algorithm are presented in Table 1, which shows the analyzed clusters 0 and 1 value in two parts. The first part shows that the total number of cluster instances assigned for cluster 0 is 138 (49%) and for cluster 1 is 143 (51%) out of 281. After the test, the number of designated clusters for NID (non-insulin dependent) diabetes attribute “type” was 128 for clusters 0 and 1. For IND (insulin dependent), it was 7 for clusters 0 and 1; for GTD (gestational diabetes patients),it was 3 for cluster 0 and 8 for cluster 1. 

The second part of the table shows the assessment results of the total number of positive tested and negative tested clusters on the diabetes dataset attribute “class.” It shows the ratios for clusters 0 and 1 for the positive and negative tests. Out of 281 instances, 47 (16.72%) are negative tested for cluster 0 and 40 (14.23%) for cluster 1. Moreover, 91 (32.38%) are positive tested for cluster 0 and 103 (36.65%) for cluster 1.

The final analysis of the first part shows that the diabetes type NID with cluster 0 and IND with cluster 1 is positive in assessment, while GTD becomes negative. In addition, the final assessment for the diabetes class attribute shows that the cluster 0 is negative tested and cluster 1 is positive tested. 

**Figure 3:**
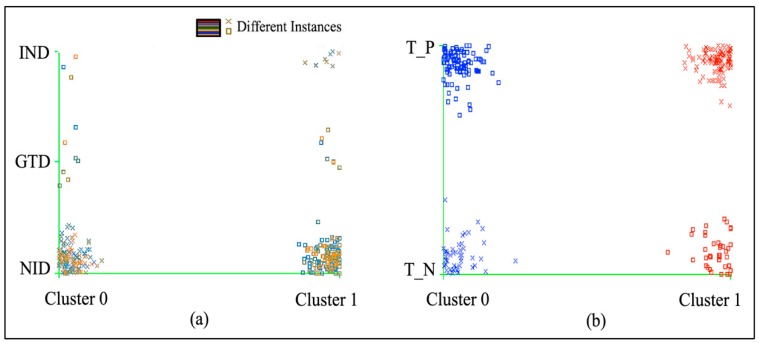
The outcomes and assessment of Table 1.

The assessment results of Table 1 are graphically illustrated in Figure 3, showing the total definition of resulted clusters on the diabetes attribute “type”and diabetes attribute “class” of the given dataset. The clusters seem to be overlaid in the graph but they are correct according to the analysis of K-mean, as described in the table to indicate the distribution of groups. 

### 3.2. SOM Assessment

After the 10-fold cross-validation execution on Weka, the self-organized map algorithm was tested on the given dataset. The model for the diabetes type attribute took 60 seconds to build and 61.07 seconds for the class attribute. The final results for the SOM algorithm are presented in Table 2, which shows the final assigned clusters of 0, 1, 2, and 3 values in two parts. The first part shows that the total number of cluster instances distributed for cluster 0 is 61 (22%), for cluster 1 is 86 (31%), for cluster 2 is 55 (20%), and for cluster 3 is 79 (28%) out of 281. After the test, the number of clusters assigned to diabetes attribute “type” for NID (non-insulin dependent) is 57 (20.28%) for cluster 0, 82 (29.18%) for cluster 1, 50 (17.79%) for cluster 2, and 67 (23.84%) for cluster 3. For IND (insulin dependent),it is 4 (1.42%) for cluster 0, 1 (0.35%) for cluster 1 and cluster 2, and 3 (1.06%) instances for cluster 3. For GTD (gestational diabetes patients), the numbers are 0 for cluster 0, 3 (1.06%) for cluster 1, 2 (0.71%) for cluster 2, and 9 (3.20%) for cluster 3. 

The second part of the table shows the assessment results of the total number of positive tested and negative tested clusters for the diabetes dataset attribute “class.” Initially, it shows the distribution ratio of instances for clusters 0, 1, 2 and 3 out of 281. After 10-fold cross-validation execution, 79 (28%) instances are distributed to cluster 0, 86 (31%) to cluster 1, 55 (20%) to cluster 2, and 62 (22%) to cluster 3. By the test implementation, the number of assigned instances to the negative tested class was 19 (6.76%) for cluster 0, 31 (11.03%) for cluster 1, 15 (5.33%) for cluster 2 and 22 (7.82%) for cluster 3. For the positive tested class, the number of assigned instances was 60 (21.35%) for cluster 0, 55 (19.57%) for cluster 1, 40 (14.23%) for cluster 2, and 39 (13.87%) for cluster 3. 

The final analysis of the first part shows that the diabetes type NID with cluster 1 and IND with cluster 0 is positive in assessment, while GTD and other chronic diseases become negative with no class status assigned. In addition, the final assessment for the diabetes class attribute shows that cluster 0 is positive tested and cluster 1 is negative tested.

**Figure 4:**
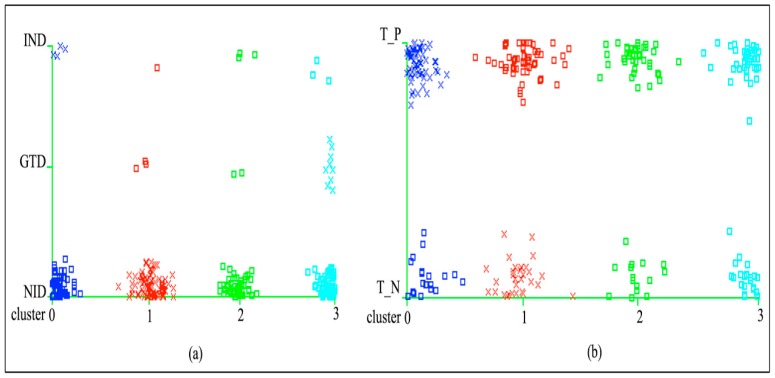
Outcomes and assessment of Table 2.

The assessment results of Table 2 are graphically illustrated in Figure 4(a) and 4(b) with the total definition of resulted clusters on the diabetes attributes “type” and “class” of the given dataset. The clusters seem to be overlaid in the graph but they are correct according to the analysis of SOM, as described in the table to indicate the distribution of groups.

**Figure 5:**
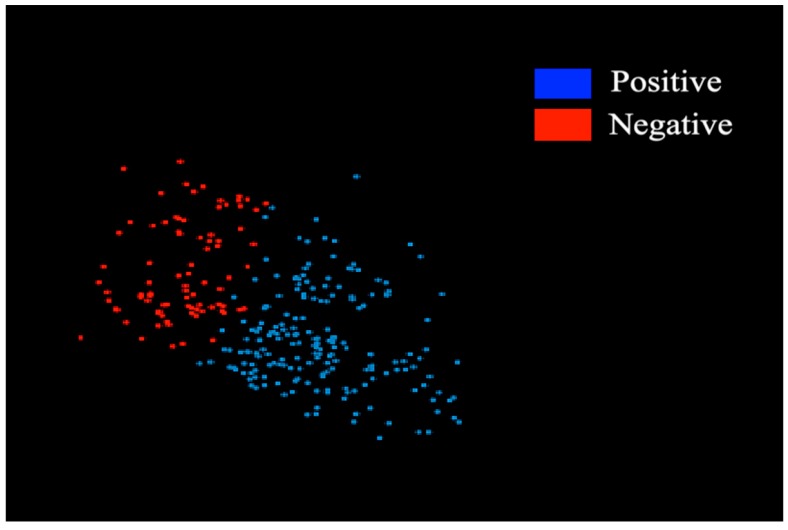
Demonstration of the graphical 2D Euclidean space illusion of the experimented dataset of diabetic patients. The Euclidean plot assessment of positive and negative clusters in the dataset of diabetes patients utilized in this research.

The projection plot in Figure 5 presents the final distribution assessment of thepositive tested and negative tested cluster instances of the diabetes dataset by class in the 2D Euclidean space. In the final assessment of machine learning algorithms, K-mean and SOM obtained 87 (30.97%) negative tested clusters and 194 (69.03%) positive tested clusters.

## 4. Discussion

This study utilizes a diabetes mellitus dataset collected only from Nigeria, containing 281 health records with 108 medical features. In this paper, both key algorithms were performed on the dataset according to the precise dimensions including the outlier actions, the distinctive shape of clusters and the functional analysis of distance. K-mean possesses an enviable record, and it performed well with a desirable analysis on the clustering to distinguish the dataset groups into positive and negative clusters. In addition, SOM has identified four cluster groups to distinguish the mellitus types in the dataset from other chronic disease symptoms. The key findings behind the SOM are the higher dimensionality of standard vectors onto a confined dimensional space. Therefore, SOM is hence regarded as a sufficient guard of topologies to the data, although K-mean also clusters to similar data points. Ultimately, the exemplification is difficult to predict because its structure needs to be amended for the social suitability.

The clustering projection plot is important for presenting the separation of positive tested and negative tested clusters in the Euclidean space. From the literature, we found that to project the cluster by machine learning algorithms, there are presently few methodologies which can collect the diverse results for the prominent approaches. We performed an extensive analysis of two machine-learning clustering algorithms (K-mean and SOM) on the real-life dataset of diabetes patients to achieve the two possible ways of obtaining desired results: one is to distinguish the related group of mellitus patients from the chronic diseases and the second is to verify the positive and negative groups of clusters. In this scenario, our approach lies within the most comprehensive phase of studies between K-mean and SOM clustering algorithms by showing the results and accurate simulation, which allows the identification of a scheme for both algorithms and also differentiation among them. Noticeably, the results of the Euclidean clustering projection plot demonstrate effectively the conclusion of simulations. 

Among the key assumptions about SOM, the first and the most important is that it is less horizontal to the local optima than K-mean. During the research evaluation and extensive experimentation, this is noticeable; SOM discovered the search space in a more effective way than K-mean. This is the desired result of vicinity parameters, which focus on units to develop according to each other in the initial method phase. Besides this, K-mean positioning potencies are impulsive in convergence, which depends on modifying that may instantly yield the finest elucidations. 

While the results of the study advocate that these two algorithms considered here worked well for the data, it is still the case that secondary analysis can be performed for the metadata in forthcoming studies. However, it is a good approach for the copious amounts of data to separate the groups by desired order. It is possible to directly modify the data before conducting the experiment, which can alter the desired results to vary promptly. These ample prospects of determined assessment are efficiently generated for future contemplations. However, we could sincerely believe that these algorithms can demonstrate and distinguish the evaluations for the massive datasets. 

## 5. Conclusions

This comprehensive study suggests that SOM is implemented more successfully than K-mean, based on the performance measurement of a few practical considerations such as the considerable number of clusters, mapping structure, error rate, computation time, involvedness and finishing time. However, after all, SOM and K-mean allow the minimization of considerable distance between the interpretations and the cluster centers. Hence, future work can be focused on the reduction of time complexity by acknowledging the cluster potentials. This assessment study is important for the initial phase of epidemiological classification of disease diagnosis such as diabetes, cancer, heart stroke rate, etc. Each classification has to go through the clustering assessment to group the likely clusters for better accuracy. Our study is particularly important for countries with higher epidemic risks and lower socioeconomic status.

## Figures and Tables

**Table 1:** The successful outcomes and consideration assessment of clusters for patient variable “DTYP” (diabetes type attribute) and “class” by privileged diabetes dataset.

**Clustering analysis on attribute “DTYP” with 9 iterations in 0.21 seconds**					
**Cluster instances**		**Assigned clusters**			**Comments**
**0**	**1**	**0**	**1**	**DTYP**	
138	143	128	128	NID	Cluster 0 is NID Cluster 1 is IND
		7	7	GTD	
49 %	51 %	3	8	IND	
**Clustering analysis on attribute “class” with a total number of 8 iterations**					
**Assigned to the cluster**					**Comments**
**0**		**1**	**Test**		
47		40	−Ve		Cluster 0 is N.T Cluster 1 is P.T^1^
91		103	+ Ve		

^1^ DTYP= diabetes type attribute; NID= not insulin dependent; IND= insulin dependent; GTD= gestational diabetes; %= considerable percentage; N.T= negative tested; and P.T= positive tested.

**Table 2:** The successful outcomes and consideration assessment of clusters for patient variables “class” and “type” by privileged diabetes status.

**Clustering analysis on attribute “DTYP” in the 60.0 seconds-built model**									
**Cluster instances**				**Assigned clusters**					**Comments**
**0**	**1**	**2**	**3**	**0**	**1**	**2**	**3**	**DTYP**	
61	86	55	79	57	82	50	67	NID	Cluster 0 is IND Cluster 1 is NID Cluster 2 has no class Cluster 3 has no class
22%	31%	20%	28%	0	3	2	9	GTD	
				4	1	3	3	IND	
**Clustering analysis on attribute “class” in the 61.07 seconds-built model**									
**Cluster instance**				**Assigned to class**					**Comments**
**0**	**1**	**2**	**3**	**0**	**1**	**2**	**3**	**class**	
79	86	55	61	19	31	15	22	N.T	Cluster 0 is P.T Cluster 1 is N.T Cluster 2 has no class Cluster 3 has no class ^2^
28%	31%	20%	22%	60	55	40	39	P.T	

^2^ DTYP= diabetes type attribute; NID= not insulin dependent; IND= insulin dependent; GTD= gestational diabetes; %= considerable percentage; N.T= negative tested; and P.T= positive tested.
